# Gamma-Aminobutyric Acid Transporters in the Nucleus Tractus Solitarii Regulate Inhibitory and Excitatory Synaptic Currents That Influence Cardiorespiratory Function

**DOI:** 10.3389/fphys.2021.821110

**Published:** 2022-01-14

**Authors:** Diana Martinez, Ludmila Lima-Silveira, Michael P. Matott, Eileen M. Hasser, David D. Kline

**Affiliations:** ^1^Department of Biomedical Sciences and Dalton Cardiovascular Research Center, University of Missouri, Columbia, MO, United States; ^2^Department of Medical Pharmacology and Physiology, University of Missouri, Columbia, MO, United States

**Keywords:** synaptic transmission, glutamate, GABA, autonomic nervous system, sympathetic nervous system

## Abstract

The brainstem nucleus tractus solitarii (nTS) processes and modulates the afferent arc of critical peripheral cardiorespiratory reflexes. Sensory afferents release glutamate to initiate the central component of these reflexes, and glutamate concentration is critically controlled by its removal *via* astrocytic neurotransmitter transporters. Gamma-aminobutyric acid (GABA) is the primary inhibitory neurotransmitter in the nTS providing tonic and phasic modulation of neuronal activity. GABA is removed from the extracellular space through GABA transporters (GATs), however, the role of GATs in nTS synaptic transmission and their influence on cardiorespiratory function is unknown. We hypothesized that GATs tonically restrain nTS inhibitory signaling and given the considerable nTS GABA-glutamate cross-talk, modify excitatory signaling and thus cardiorespiratory function. Reverse transcription real-time polymerase chain reaction (RT-PCR), immunoblot and immunohistochemistry showed expression of GAT-1 and GAT-3 mRNA and protein within the rat nTS, with GAT-3 greater than GAT-1, and GAT-3 colocalizing with astrocyte S100B. Recordings in rat nTS slices demonstrated GAT-3 block decreased spontaneous inhibitory postsynaptic current (IPSC) frequency and reduced IPSC amplitude evoked from electrical stimulation of the medial nTS. Block of GAT-3 also increased spontaneous excitatory postsynaptic current (EPSC) frequency yet did not alter sensory afferent-evoked EPSC amplitude. Block of GAT-3 in the nTS of anesthetized rats increased mean arterial pressure, heart rate, sympathetic nerve activity, and minute phrenic nerve activity. These results demonstrate inhibitory and excitatory neurotransmission in the nTS is significantly modulated by endogenous GAT-3 to influence basal cardiorespiratory function.

## Introduction

The brainstem nucleus tractus solitarii (nTS) is the first central site for integration and modulation of multiple cardiorespiratory sensory reflexes and is critical for autonomic homeostasis. Sensory afferent processing is primarily initiated by the excitatory neurotransmitter glutamate (Kline, 2008) and such excitation is counter-balanced through gamma-aminobutyric acid (GABA) inhibition. GABA interneurons are located throughout the nTS (Izzo et al., 1992; Fong et al., 2005) and release GABA that mediates both rapid phasic and prolonged inhibition *via* ionotropic GABA_*A*_ receptors and metabotropic GABA_*B*_ receptors respectively (Mehta and Ticku, 1999; Ulrich and Bettler, 2007). Activation of GABA receptors in the rat nTS increases heart rate (HR) and blood pressure, blunts the baroreflex (Catelli et al., 1987; Sved and Sved, 1990), and decreases basal respiration and the hypoxic ventilatory response (Tabata et al., 2001; Suzuki et al., 2003).

We have previously demonstrated astrocytes within the nTS tripartite synapse critically control glutamate signaling and cardiorespiratory baseline and reflex function (Matott et al., 2016, 2017; Martinez et al., 2020b). Such modulation occurs *via* the tonic modulation of glutamate in the extracellular space *via* astrocytic excitatory amino acid transporters (EAATs), which play important roles in synaptic transmission and its alterations in response to prolonged hypoxia (Martinez et al., 2020a; Matott et al., 2020). While not established in the nTS, similar to glutamate signaling, modulation of GABA signaling and overall inhibitory tone occurs *via* GABA transporters (GATs; Zhou and Danbolt, 2013). GAT-1 and GAT-3 are the primary GATs in the brain and spinal cord (Zhou and Danbolt, 2013). GAT-1 is heterogeneously expressed in neuronal terminals and astrocytes, whereas GAT-3 is primarily observed in astrocytes (Ng and Ong, 2001; Park et al., 2009; Zhou et al., 2012; Yadav et al., 2015). Within the paraventricular nucleus of the hypothalamus, GATs tonically limit GABA inhibitory postsynaptic currents (IPSCs), modulate membrane properties, action potential discharge (APd), and sympathetic activity (Park et al., 2009), suggesting GABA signaling is basally limited by its endogenous uptake. Support for a role of GATs within the nTS is indicated by the increases in blood pressure and apneustic breathing following general block of GABA uptake with nipecotic acid (Catelli et al., 1987; Wasserman et al., 2002), but the influence of nTS GATs is relatively unknown. GABA also influences glutamatergic signaling in the nTS where it may increase or decrease excitation (Fernandes et al., 2011; Kang et al., 2012; McDougall and Andresen, 2012). Given the importance of GABAergic signaling in the nTS, GATs are likely to contribute importantly to nTS synaptic and neuronal modulation, GABAergic and glutamatergic balance, and cardiorespiratory function. We hypothesized that GATs tonically restrain nTS GABA tone to balance inhibition and excitation thereby influencing baseline cardiorespiratory function. To test this hypothesis, we determined the expression and synaptic and physiological roles of GATs, primarily GAT-3 in the caudomedial nTS.

## Materials and Methods

### Ethical Approval

Experiments were performed on four to eight-week-old male Sprague-Dawley rats (Envigo, Indianapolis, IN, United States). Animals were housed in a 12:12-h light/dark cycle with water and food available *ad libitum*. Experiments were performed following the National Institutes of Health’s *Guide for the Care and Use of Laboratory Animals* guidelines. Animal protocols were approved by the University of Missouri Animal Care and Use Committee.

### Reverse Transcription Real-Time Polymerase Chain Reaction

Rats (*N* = 6) were anesthetized with isoflurane, rapidly decapitated and the caudal portion of the medial and commissural nTS divisions bound by the area postrema and solitary tract (i.e., caudomedial nTS) was isolated. The nTS was then flash frozen and stored at −80°C until ready for use. RNA was isolated using the RNAqueous-Micro kit, following the manufacturer’s instructions (Ambion #AM1931, Life Technologies, Grand Island, NY, United States), and quantified (BioPhotometer Plus; Eppendorf, Hauppauge, NY, United States). cDNA was generated using 100 ng of mRNA (oligo-dT primer set, SuperScript III; Invitrogen #18080-051). Quantitative real-time PCR amplification of 2 μL of cDNA was performed using the SYBR Premix Ex Taq kit (Takara, Mountain View, CA, United States), the SmartCycler System (Cepheid, Sunnyvale, CA, United States), and the following primers: *GAT-1* [NM_024371.1 (forward: AGA GGT CCA TAG CCG ATG TG, reverse CTC GGG GTA TGC CAA GAA T:)], *GAT-3* [NM_024371.1 (forward: CTC CCG GCT CTC TGA TCC, reverse: GGC AGA TGG CAT AGG AGA AA)], and the housekeeping gene β_2_-microglobulin [(*B2m*, NM_0122512.2) (forward: AGC AGG TTC CTC AAA CAA GG, reverse: TTC TGC CTT GGA GTC CTT TC, 10 μM; Fisher Scientific)]. The relative quantity of GAT-1 and GAT-3 mRNA was normalized to *B2m* using the 2^–Δ^ ^Δ^ *^CT^* method (Livak and Schmittgen, 2001).

### Immunoblot Analysis

Immunoblot analysis was used to determine the relative concentration of GAT-1 and GAT-3 protein. Rats (*N* = 5) were anesthetized and decapitated and the nTS quickly removed. The caudomedial nTS from each rat was isolated as above and homogenized in extraction buffer [150 mM NaCl, 100 mM Tris–HCl, 1% Triton X, protease inhibitor cocktail (Complete Mini, EDTA-free; Catalog #1183617001, Roche Diagnostics, Indianapolis, IN, United States)], centrifuged (10 min, 13,000 rpm, 4°C), and the supernatant was collected.

The Bio-Rad Protein Assay Dye Reagent (Bio-Rad, Hercules, CA, United States) was used to determine protein concentration. Protein from each rat (20 and 40 μg) were separated in a 4–20% gel and transferred to an Immun-Blot polyvinylidene difluoride membrane (Bio-Rad). Membranes were then incubated overnight at 4°C with primary antibodies against GAT-1 (1:500, Millipore #AB1570W, RRID:AB_90791), GAT-3 (1:500, Millipore #AB1574, RRID:AB_90779), and tubulin (1:1000; Abcam #ab7291, RRID:AB_2241126). After this incubation period, the membrane was washed and incubated with horseradish peroxidase-linked secondary antibodies (1:10,000 Jackson ImmunoResearch Laboratories). Blots were developed using ImmunStar WesternC substrate (Bio-Rad) and imaged with the ChemiDoc XRS + Imager using Image Laboratory Software (version 5.1; Bio-Rad). Intensity of bands were measured using ImageJ; relative amounts of GAT-1 and GAT-3 were normalized to tubulin and quantified.

### Immunohistochemistry

Isoflurane anesthetized rats (*N* = 3) were transcardially perfused with ice-cold 0.01 M phosphate-buffered saline (PBS; 125 mL) followed by 4% paraformaldehyde (250 mL, pH = 7.4; Sigma). Brains were post-fixed overnight in 4% paraformaldehyde, and then stored in 0.01 M PBS until use. The brainstems were removed and coronally sectioned using a vibratome (VT1000S, Leica, 30 μm). Sections were rinsed in 0.01 M PBS (3 × 10 min) and then blocked with 10% Normal Donkey Serum (NDS; S30; Millipore) in 0.3% Triton-PBS for 1 h. Tissue sections were then rinsed with 0.01 M PBS (3 × 10 min) and subsequently incubated with one or more of the following primary antibodies: GAT-1 (1:750, Synaptic Systems #274 102, RRID:AB_2620000) or GAT-3 (1:750, Millipore #AB1574, RRID:AB_90779), and GAD-67 (enzyme for GABA synthesis, 1:5000, Chemicon #MAB5406, RRID:AB_2278725) in 3% NDS in 0.01 M PBS. To further identify astrocytes, we used S100B (1:1000, Synaptic Systems, #287 004, RRID:AB_2620025) to label astrocytic processes. Following incubation, sections were rinsed and incubated for 2 h in the appropriate secondary fluorescent antibody in 3% NDS and 0.3% Triton-PBS. Sections were rinsed, mounted on gelatin-coated slides, allowed to air dry, and coverslipped. Slides were then sealed with clear nail polish. One section per run was incubated without primary antibody and served as a negative control. No fluorescent staining was present on negative controls.

Immunoreactivity within the medial nTS was examined with a Nikon Eclipse Ti2 microscope equipped with a 60X oil objective (NA 1.4), Yokakawa CSU-W1 SoRa confocal scanner unit, and Hamamatsu Orca-Fusion CMOS camera (C14440-20UP). For each fluorophore used, *z*-stacks (0.15 μm between images) were taken in the same focal planes. Under this configuration the resolution was 120 nm. Images were deconvolved using Nikon’s NIS-Elements software (20 iterations), and postprocessed for contrast and brightness (ImageJ; National Institutes of Health, RRID:SCR_003070).

### Brain Slice Electrophysiology

The brainstems of isoflurane-anesthetized rats were quickly removed and placed into an ice cold NMDG-HEPES cutting solution (in mM: 93 NMDG, 2.5 KCl, 1.2 NaH_2_PO_4_, 10 MgSO_4_, 30 NaHCO_3_, 20 HEPES, 25 D-glucose, 5 L-ascorbic acid, 2 thiourea, 3 sodium pyruvate, and 0.5 CaCl_2_, aerated with 95% O_2_/5% CO_2_, pH to 7.4 with ∼93 HCl, and 300–310 mosM). Horizontal slices (∼280 μm) were cut using a vibratome (VT1200S, Leica). The slices were allowed to recover for 12 min in the NMDG-HEPES solution (32°C) and then placed into standard recording aCSF (in mM: 124 NaCl, 3 KCl, 1.2 NaH_2_PO_4_, 1.2 MgSO_4_, 25 NaHCO_3_, 11 D-glucose, and 2 CaCl_2_, saturated with 95% O_2_/5% CO_2_, pH 7.4, and ∼300 mosM) at 31–33°C for 30 min.

For recording, slices were placed in a superfusion chamber, secured *via* nylon mesh, and superfused at ∼2–3 mL/min with recording aCSF. All recordings were made from somas in the caudomedial nTS, which has been shown to receive cardiorespiratory afferent input (Kline, 2008) and corresponds to the region where nanoinjections were made (Ostrowski et al., 2014). Recordings were acquired in pClamp 10.7 using a Multiclamp 700B amplifier (Molecular Devices) and filtered at 2 kHz. Neurons were excluded if the membrane potential was depolarized more than −45 mV upon initial break-in, or if the series resistance was greater than 25 MΩ, or changed 20% or more throughout the experiment. We examined the effect of blocking GAT-3 *via* application of (S)-1-[2-[Tris(4-methoxyphenyl)methoxy]ethyl]-3-piperidinecarboxylic acid [SNAP 5114, 50 μM, 5 min (Wu et al., 2014; Moldavan et al., 2017)] on synaptic and membrane events. For evoked events, stimulation parameters encompassed the physiological discharge range of sensory afferents (Andresen and Kunze, 1994; Kline, 2008).

#### Inhibitory Postsynaptic Currents

Gamma-aminobutyric acid-mediated IPSCs were recorded *via* glass pipettes filled with (in mM) 140 CsCl, 5 NaCl, 10 EGTA, 10 HEPES, 1.2 MgSO_4_, 3 K-ATP, 0.2 Na-GTP, 5 QX314, pH 7.3, and ∼280 mosM. This internal solution and use of recording aCSF (above) generated a reversal potential of chloride of ∼3 mV. Cells were held at −60 mV and aCSF contained the glutamate receptor blockers NBQX (10 μM, AMPA receptor blocker; Tocris) and AP5 (10 μM, NMDA receptor blocker; Tocris) (Ostrowski et al., 2014; Matott et al., 2017). This configuration produced inward currents that were easily identified and eliminated by the GABA_*A*_ receptor blocker GABAzine (Ostrowski et al., 2014). Spontaneous (s) IPSCs were recorded in gap-free mode and acquired at a sampling rate of 20 kHz. nTS-evoked IPSCs were generated with a concentric bipolar stimulating electrode placed on the nTS neuropil and evoked *via* an isolated stimulator (100 μs, 10–200 μA, 0.5 Hz, A.M.P.I., Master-8 and ISO-Flex). Sampling rate for nTS-IPSCs evoked at 0.5 Hz were acquired at 100–200 kHz, whereas repetitive currents for paired-pulse analysis were evoked at 20 Hz and sampled at 20 kHz.

#### Excitatory Postsynaptic Currents

Glutamate-mediated excitatory postsynaptic currents (EPSCs) were recorded with pipettes filled with (in mM) 10 NaCl, 130 K^+^-gluconate, 11 EGTA, 1 CaCl_2_, 10 HEPES, 1 MgCl_2_, 2 Mg-ATP, 0.2 Na-GTP, pH 7.3, and ∼280 mosM. Cells were held at −60 mV. Chloride reversal potential in these recordings was ∼−62 mV. Spontaneous (s) EPSCs were recorded in gap-free mode and acquired at a 20 kHz sampling rate. Afferent (TS, solitary tract) evoked EPSCs were induced at 0.5 and 20 Hz by stimulating the TS for 100 μs, at 10–200 μA *via* a concentric electrode. Solitary-tract evoked excitatory postsynaptic currents (TS-EPSCs) were acquired at a sampling rate of 250 kHz when evoked at 0.5 Hz, or 20 kHz for events evoked at 20 Hz.

#### Membrane Potential and Action Potential Discharge

Membrane properties utilized the same extracellular and intracellular solutions as described for EPSCs. Membrane potential and spontaneous APd were recorded in *I* = 0 mode (no current added). APs were also evoked *via* current depolarization (10 pA steps, 100 ms).

#### *In vitro* Analysis

Electrophysiological data were analyzed using Clampfit 10.7 (Molecular Devices) and MiniAnalysis Program (RRID:SCR_002184). Electrophysiological properties were acquired and analyzed following the initial 5 min aCSF baseline, 5 min of GAT-3 block (SNAP 5114) and 5 min of wash. Each cell and slice were exposed to SNAP 5114 only once. Spontaneous events were measured in 30 s periods. The amplitude, area and decay tau (90–10%) of 0.5 Hz stimulus-evoked currents were determined by averaging 20 individual events. For 20 Hz TS-EPSCs, a train of ten events was measured across 5 episodes and averaged. For 20 Hz nTS-IPSCs, the amplitude ratio of the second event was compared to the first event (paired pulse ratio; Kline et al., 2002). Membrane potential (V_*m*_) was monitored in 30 s periods, and spontaneous APd measured in 60 s periods.

### *In vivo* Cardiorespiratory Parameters

*In vivo* experiments were performed as previously (Ostrowski et al., 2014; Matott et al., 2017; Ruyle et al., 2019). Rats (*N* = 6) were anesthetized with isoflurane (5%, induction; 2–2.5% maintenance, in 100% O_2_), tracheotomized and mechanically ventilated with O_2_-enriched room air. Arterial blood gases were periodically measured (Osmetech, OPTI CCA Roswell, GA, United States) and tidal volume or respiratory rate adjusted as necessary to maintain blood gases. Temperature was monitored rectally and maintained at ∼38°C.

Arterial and venous femoral catheters (PE-10 fused to PE-50, A-M Systems, Sequim, WA, United States) were implanted to measure arterial pressure and HR, and to allow administration of fluids or drugs. Using a ventral cervical approach, the right phrenic nerve (PhrN) was isolated, placed on a bipolar recording electrode (Teflon-coated silver wire, 0.005″ A-M Systems), and covered in a silicone elastomer (Kwik-Cast, WPI Sarasota, FL, United States) to record its activity (i.e., PhrNA). The left phrenic nerve was severed, and the right was distally crushed. Similarly, *via* a retroperitoneal approach the left splanchnic nerve was isolated to record splanchnic sympathetic nerve activity (SSNA). In order to prevent entrainment of phrenic motor output with the ventilator rats were subjected to bilateral cervical vagotomy. Ground wires were sutured to the surrounding muscle to reduce noise, and incisions were closed. SSNA and PhrNA were amplified (1000×), filtered (30–3000 Hz, P511, Grass technologies, Warwick, RI, United States), rectified and integrated (time constant: phrenic = 100 ms; splanchnic = 28 ms) using a root mean square converter (PowerLab data acquisition system, Version 7; ADInstruments, Colorado Springs, CO, United States, RRID:SCR_001620). SSNA was electronically averaged. Background noise in both nerves was determined from the signal between bursts of activity and verified following euthanasia. SSNA and PhrNA were defined as the appropriate recorded nerve activity minus background noise. The pulsatile arterial pressure signal was used to determine mean arterial pressure (MAP) and HR (PowerLab).

Following nerve isolation, rats were placed in a stereotaxic apparatus (Kopf Instruments, Tujunga, CA, United States) and the dorsal surface of the brainstem exposed *via* a partial occipital craniotomy. Once surgery was complete, anesthesia was converted over 30 min from Isoflurane to Inactin (100 mg/kg i.v., with 5 mg/kg i.v. supplements as needed). Animals were subjected to neuromuscular blockade with gallamine (12.5 mg/kg i.v., 3–4.5 mg/h i.v. maintenance). Adequate plane of anesthesia was regularly verified by lack of cardiovascular responses (<5 mmHg increase in MAP) to a firm tail pinch. Ventilation with O_2_-enriched room air was maintained above apneic threshold by adjusting tidal volume and breathing frequency. A period of ∼60 min was allowed for cardiorespiratory parameters to stabilize before initiating any experimental procedures. Glass micropipettes (1–2 barrels, ∼10 μm per barrel, borosilicate glass, WPI) filled with the GAT inhibitor SNAP 5114 (1 mM) were advanced into the caudomedial nTS under visual guidance. Four nanoinjections (45–90 nL) were targeted to the caudal medial and commissural nTS [relative to calamus scriptorius (CS; mm): 0 and 0.4 anterior, 0.2 and 0.4 lateral, and 0.4 ventral to the dorsal surface of the medulla] and comparable to *in vitro* recordings (Ostrowski et al., 2014). MAP, HR, SSNA, and PhrNA were monitored continuously during experimental GAT-3 manipulation. Minute phrenic activity (Min PhrNA) was defined as the product of phrenic frequency (Phr Freq) and amplitude (Phr Amp). SSNA responses were evaluated as a percentage of baseline activity prior to a given intervention.

### Reagents

SNAP 5114 (GAT-3 blocker), NBQX (AMPAR blocker) and AP5 (NMDAR blocker) were purchased from Tocris Bioscience (R&D Systems, Inc., Minneapolis, MN, United States). Stocks of 100 mM SNAP 5114, 100 mM NBQX and 10 mM AP5 were made in DMSO or double distilled water and diluted in aCSF to reported concentrations. All other chemicals were purchased from Sigma−Aldrich (St. Louis, MO, United States) and Fisher Scientific (Pittsburgh, PA, United States).

### Statistical Analysis

Statistical analysis occurred *via* GraphPad Prism 9 or Microsoft Excel software. Reverse transcription real-time polymerase chain reaction (RT-PCR) and immunoblots examining GAT-1 vs GAT-3 (two groups) within an individual were compared using a paired two-tailed *t*-test. For *in vitro* electrophysiology (e.g., IPSC amplitude, area, and decay), following confirmation of normality, the overall effects of SNAP 5114 relative to its initial aCSF baseline and following washout (three groups) were tested using one-way repeated measures (RM) ANOVA or mixed-effects analysis. When sphericity was not assumed based on unequal variability of differences the Geisser and Greenhouse correction was used [epsilon (ε) reported]. When two groups with repeats were examined (i.e., TS-EPSC amplitude during 20 Hz stimulus train and APd in response to current injection) a two-way RM ANOVA was used. LSD *post hoc* analysis examined specific differences following ANOVA. For data sets not normally distributed, a Kruskal–Wallis test was used followed by an uncorrected Dunn’s multiple comparison test. Cardiorespiratory parameters between baseline and SNAP 5114 (two groups) were evaluated using a paired two-tailed *t*-test. “*N*” denotes number of rats whereas “*n*” indicates number of cells. *p* ≤ 0.05 was considered significant for all tests. Data are presented as mean ± standard error of the mean (SEM).

## Results

### Gamma-Aminobutyric Acid Transporter 1 and 3 Are Present in the Nucleus Tractus Solitarii and Associated With Astrocytes

**FIGURE 1:**
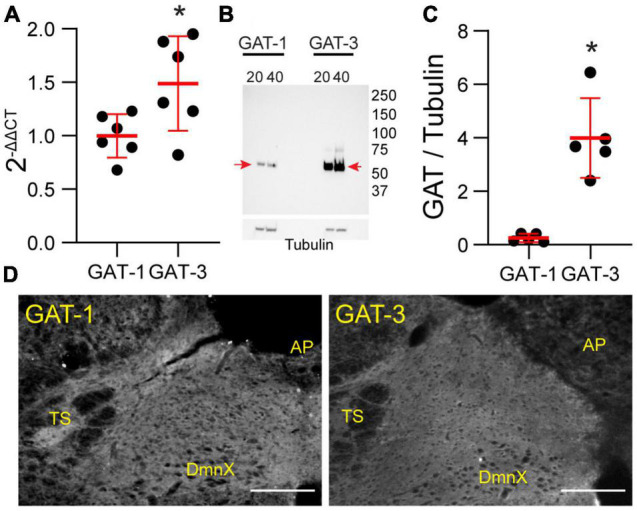
Gamma-aminobutyric acid transporters 1 and 3 are expressed in the nTS. **(A)** Quantification of mRNA transcript of GAT-1 and GAT-3 in the nTS. GAT-3 mRNA transcript was greater than in GAT-1 (*N* = 6, *p ≤ 0.05 paired *t*-test). **(B)** Immunoblot example GAT-1 and 3 in the nTS [*left*, GAT-1 (20 and 40 μg) and *right*, GAT-3 (20 and 40 μg)]. Note the GAT-1 band at ∼67 and the GAT-3 band at ∼70 kDa. Tubulin was used as a control. **(C)** Quantification of the GAT-1/tubulin ratio (*left*) and GAT-3/tubulin ratio (*right*). GAT-3 is found in higher quantities in the nTS than GAT-1. *N* = 5, *p ≤ 0.05 paired *t*-test. **(D)** IHC of GAT-1 and GAT-3 in nTS. TS, solitary tract; DmnX, dorsal motor nucleus of the vagus, AP, area postrema. Scale, 200 μm.

**FIGURE 2:**
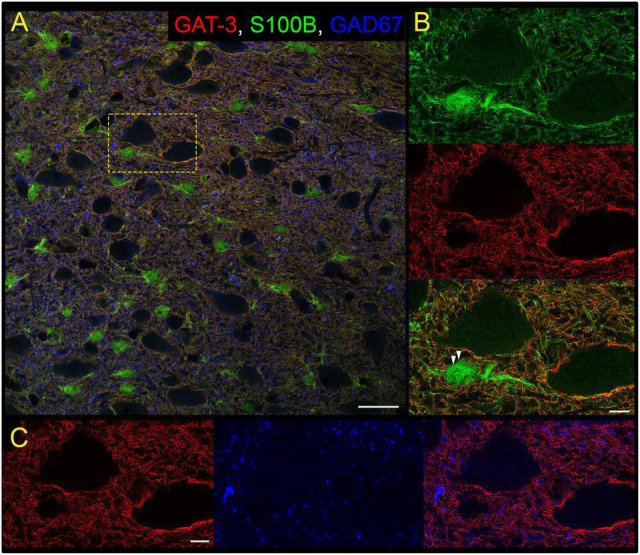
Gamma-aminobutyric acid transporter 3 is found on nTS astrocytes. **(A)** Overview of immunoreactivity of GAT-3 (red), astrocyte S100B (green) and GABAergic GAD67 terminals (blue). Dashed box is shown in **(B,C)**. Scale, 25 μm. **(B)** Super-resolution image of S100B and GAT-3, and their overlay, demonstrating GAT-3 aligns with S100B. Arrowheads denote GAT-3 on the membrane of S100B-labeled astrocyte somas. Scale, 5 μm. **(C)** GAT-3 surrounds GAD67-labeled GABA terminals (arrow). Scale, 5 μm. Shown are single optical sections.

Our study focused on the contribution of GATs in the medial nTS. The expression of GAT-1 and GAT-3 in the caudomedial nTS was examined at the mRNA transcript and protein level. RT-PCR confirmed expression of GAT-1 and GAT-3 mRNA, with relatively greater expression of GAT-3 compared to GAT-1 (Figure 1A, paired *t*-test, *N* = 6). Immunoblot analysis of protein expression confirmed greater expression of GAT-3 relative to GAT-1. A representative immunoblot (Figure 1B) of 20 and 40 μg nTS protein from one individual rat demonstrates single bands for GAT-1 (∼67 kDa) and GAT-3 (∼70 kDa) at the appropriate molecular weights, and greater intensity of bands for GAT-3. Quantitatively, GAT-3 was greater than GAT-1 in the nTS (20 μg, Figure 1C, paired *t*-test, *N* = 5, a 16 ± 6-fold increase). Similarly, a 19 ± 10-fold increase of GAT-3 over GAT-1 was observed when compared at 40 μg (*p* = 0.014, paired *t*-test). GAT-1 and GAT-3 immunoreactivity was observed throughout the caudomedial nTS as diffuse staining with similar observations for both proteins (Figure 1D). Given the greater expression of GAT-3, we further confirmed its co-labeling with the glial-marker S100B (Figures 2A,B) and its proximity to GAD67 (GABAergic) terminals (Figure 2C). Due to the greater expression of GAT-3 to GAT-1, we focused on the influence of GAT-3 in the nTS circuit and overall cardiorespiratory and autonomic function.

### Gamma-Aminobutyric Acid Transporter 3 Tonically Controls Spontaneous and Evoked Inhibitory Postsynaptic Currents

**FIGURE 3:**
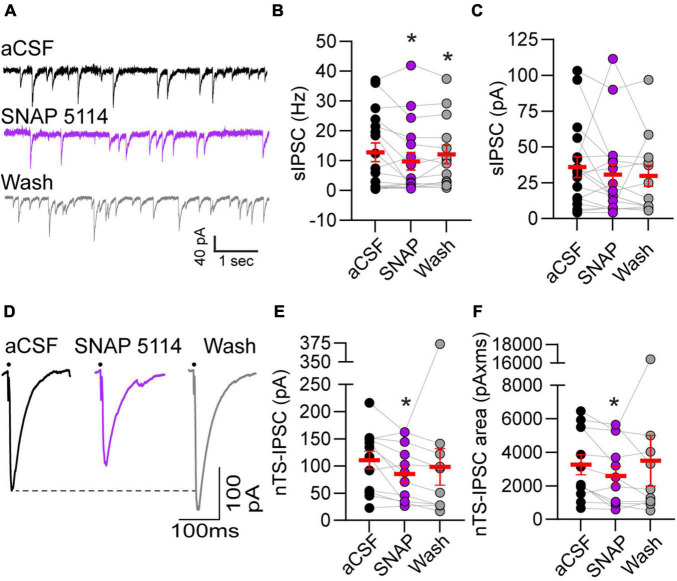
Gamma-aminobutyric acid transporter 3 blockade reduced spontaneous and evoked IPSCs. **(A)** Representative example of spontaneous IPSCs under baseline aCSF, in the presence of GAT-3 antagonist SNAP 5114 and in aCSF wash. All recordings were done in the presence of the AMPA and NMDA receptor blockers NBQX and AP5, respectively, in the aCSF and a high chloride intracellular solution to produce inward GABAergic currents. Note the reduction in frequency with GAT-3 blockade. Quantitatively, sIPSC frequency was decreased **(B)** but amplitude was unchanged **(C)** suggesting pre-synaptic reduction in GABA release. *n* = 17. Mixed-effects analysis with LSD *post hoc*: **(B)** *F*(2,29) = 3.619; **(C)** *F*(2,29) = 0.4792, *p ≤ 0.05 vs aCSF. **(D)** Representative trace of nTS-IPSCs evoked at 0.5 Hz. Dots denotes time of nTS-stimulation (artifacts removed for clarity). GAT-3 block decreased nTS-IPSC amplitude and area compared to aCSF baseline, which is quantified in **(E,F)** (*n* = 12). Mixed-effects analysis with Geisser–Greenhouse correction and LSD *post hoc*: **(E)** *F*(1.331,13.31) = 1.130, ε = 0.6654; **(F)** *F*(1.064,10.64) = 0.5885, ε = 0.5319, *p ≤ 0.05 vs aCSF. Data in **(B,C,E,F)** are shown as individual neurons (connected circles), their responses, and means ± SEM in red.

Astrocytic GATs serve to remove GABA from the extrasynaptic space. To examine the extent endogenous GAT-3 modulates GABA receptor-mediated signaling we analyzed IPSCs using a high-chloride intracellular solution and glutamate AMPA and NMDA receptor blockers. As shown in the representative traces (Figure 3A), bath application of the GAT-3 blocker SNAP 5114 (5 min) decreased the frequency of spontaneous IPSCs from its aCSF baseline. Mean data show GAT-3 inhibition decreased sIPSC frequency from baseline, and events remained reduced 5 min following SNAP 5114 washout (Figure 3B, *n* = 17, *N* = 14). However, sIPSC amplitude was not altered by GAT-3 block comparable to its aCSF baseline (Figure 3C, *n* = 17, *N* = 14).

Gamma-aminobutyric acid release was evoked *via* stimulation of the nucleus tractus solitarii neuropil at 0.5 Hz and the resulting currents examined (i.e., nTS-IPSCs). As shown in the example traces in Figure 3D, nTS-IPSC amplitude decreased with SNAP 5114-mediated GAT-3 block. Quantitatively, nTS-IPSC amplitude and area significantly decreased in SNAP 5114 relative to aCSF baseline (Figures 3E,F, *n* = 12, *N* = 11). However, nTS-IPSC decay (90-10%) was not altered by GAT-3 block [aCSF, 19.5 ± 2.7 ms; SNAP 20.5 ± 3.3 ms; wash, 24.4 ± 4.3 ms; *n* = 12, *N* = 11, mixed-effects analysis, *F*(2,20) = 2.023, *p* > 0.05]. To examine the potential site of SNAP-induced changes, we stimulated the nTS twice in succession separated by 50 ms (20 Hz). The amplitude ratio of the second to first nTS-IPSC was examined. Block of GAT-3 increased the paired-pulse ratio from 0.68 ± 0.08 (aCSF) to 1.22 ± 0.26 (SNAP 5114, *n* = 7, *N* = 7, Kruskal–Wallis test, *p* = 0.067, *p* = 0.09 vs aCSF), which did not completely reverse upon washout (1.00 ± 0.07, *p* = 0.02 vs aCSF). Block of GAT-3 also produced a small significant outward shift in the holding current from aCSF (+5.2 ± 1.2 pA, *p* ≤ 0.05 vs aCSF, RM ANOVA, *n* = 13, *N* = 12) that reversed upon 5-min washout [+2.7 ± 5.0 pA, one-way RM ANOVA, *F*(1.088,13.05) = 0.8066, ε = 0.5438].

### Gamma-Aminobutyric Acid Transporter 3 Block Increases Spontaneous Excitatory Currents but Does Not Alter Afferent Driven Events

**FIGURE 4:**
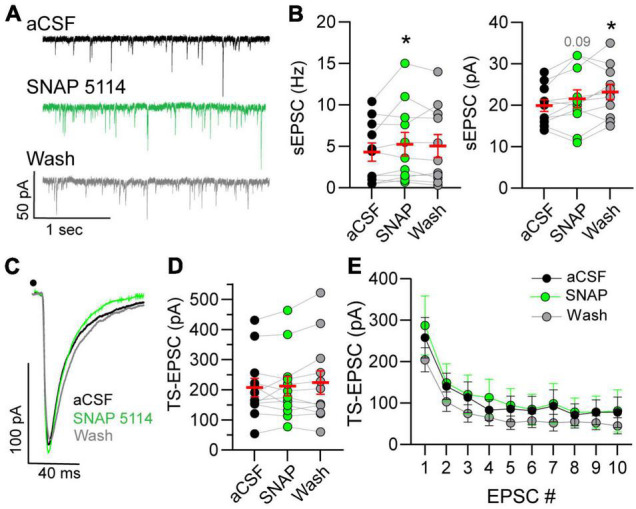
Spontaneous glutamatergic activity increases in GAT-block. **(A)** Example of spontaneous excitatory postsynaptic current (sEPSCs) increase during GAT-3 block with SNAP 5114. Recordings occur in potassium-gluconate pipette solution. **(B)** An augmentation in frequency (Hz, *left*) occurred whereas amplitude (pA, *right*) progressively increased over time. RM one-way ANOVA with LSD *post hoc*: Freq left, *F*(2,20) = 2.320; Ampl right, *F*(2,20) = 6.026, *p ≤ 0.05 vs aCSF. **(C)** Representative traces of TS-EPSCs evoked by 0.5 Hz stimulation during aCSF, SNAP 5114 and wash. Overall, TS-EPSC amplitude did not change during 0.5 **(D)** or 20 **(E)** Hz afferent stimulation in the presence of GAT-3 inhibition. **(D)** RM one-way ANOVA with LSD *post hoc*: *F*(2,22) = 1.055. **(E)** Mixed-effects model (REML), EPSC main factor, *F*(9,81) = 26.04, drug main factor, *F*(2,18) = 0.4005, interaction, *F*(18,142) = 0.8677. Data in **(B,D)** (left) are shown as individual neurons (connected circles), their responses, and means ± SEM in red. *p ≤ 0.05.

The influence of GAT-3 on glutamatergic activity is unknown, and therefore we examined the effects of GAT-3 blockade on both spontaneous and afferent-driven glutamate EPSC activity. This was accomplished *via* use of a low-chloride, potassium gluconate-based pipette solution. As shown in the representative example (Figure 4A), 5 min of SNAP 5114 application increased the frequency of spontaneous currents. sEPSC amplitude was modestly elevated during the initial 5 min of GAT-3 block and continued to increase during its washout. sEPSC frequency and amplitude are quantified in Figure 4B.

Spontaneous currents represent all inputs that impinge on the recorded neuron. This may include input not only from sensory fibers but also glutamatergic interneurons and projections from other central nuclei. To directly assess the influence of GAT-3 on the monosynaptic sensory afferent-nTS synapse, we examined TS-EPSCs in response to 0.5 and 20 Hz stimulation. The latter was examined as we reasoned that increased stimulation rate may recruit GABA and thus GAT-3 may modulate sensory release. As seen from the representative traces in Figure 4C and quantified in Figure 4D, TS-EPSC amplitudes evoked at 0.5 Hz were unaltered by GAT-3 block. In addition, GAT-3 block did not affect TS-EPSC amplitude in response to repetitive 20 Hz stimulation (Figure 4E). Block of GAT-3 produced a small inward shift in the holding current from aCSF that did not reach significance (−3.7 ± 2.7 pA, *p* = 0.17, RM ANOVA, *n* = 10, *N* = 9). After 5 min of washout, the holding current depolarized further and was significantly more depolarized than at baseline (−6.5 ± 2.2 pA, *n* = 10, *p* ≤ 0.05).

### Gamma-Aminobutyric Acid Transporter 3 Block Depolarizes Nucleus Tractus Solitarii Neurons and Increases Discharge

**FIGURE 5:**
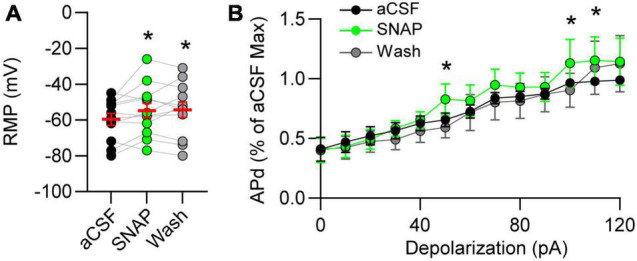
Gamma-aminobutyric acid transporter 3 influences membrane potential and discharge. **(A)** Resting membrane potential (RMP) depolarized in GAT-3 block. RM one-way ANOVA with LSD *post hoc*: *F*(2,22) = 3.543, *p ≤ 0.05 vs aCSF. **(B)** Action potential discharge (APd) evoked *via* current depolarization. Data plotted to maximum discharge of aCSF baseline (denoted as “1” at 120 pA injection). Note GAT-3 inhibition increased discharge at the higher current injection. Two-way RM ANOVA; pA main factor, *F*(12,120) = 32.08, *p* ≤ 0.001; drug main factor, *F*(1,10) = 0.8105, *p* = 0.38; interaction, *F*(12,120) = 0.8703, *p* = 0.57. *p ≤ 0.05 vs aCSF. Data in A shown as individual neurons (connected circles), their responses, and means ± SEM in red.

Resting membrane potential (RMP) is influenced by the balance of GABA and glutamate. Thus, we examined the effects of GAT block on RMP of nTS neurons and found that GAT-3 block depolarized nTS neurons in comparison to aCSF (Figure 5A). Spontaneous APd under aCSF baseline was variable at resting potential, which was not unexpected given its range (mean, −59.6 ± 3.2 mV; range, −80 to −45 mV, *n* = 12, *N* = 9), and GAT-3 block did not alter the overall number of spontaneous APs [aCSF, 28 ± 19 vs SNAP 5114, 34 ± 25, *n* = 12, *N* = 9, one-way RM ANOVA with LSD *post hoc*, *F*(2,22) = 0.9906, *p* > 0.05]. However, further analysis revealed a positive correlation between the magnitude of SNAP 5114-induced membrane depolarization and increase in spontaneous discharge (*r* = 0.65, Spearman *p* = 0.02, *n* = 12, *N* = 9). We therefore examined discharge in response to 10 pA depolarizing steps (0–120 pA). While GAT-3 inhibition overall did not increase discharge (*p* > 0.05, main effect), further examination of individual depolarizing steps revealed SNAP increased discharge at greater depolarization (Figure 5B).

### Nucleus Tractus Solitarii Gamma-Aminobutyric Acid Transporter 3 Inhibition Increases Mean Arterial Pressure, Heart Rate, Sympathetic and Phrenic Nerve Activity

**FIGURE 6:**
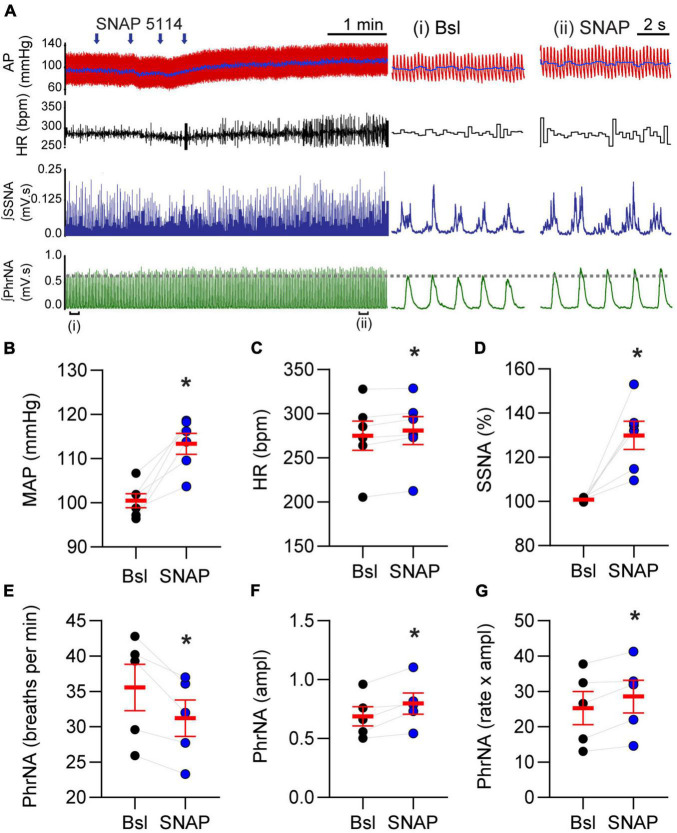
Gamma-aminobutyric acid transporters prevent increases in blood pressure, heart rate, sympathetic nerve activity and phrenic nerve discharge. **(A)** *Left*: Example of cardiorespiratory response to nTS nanoinjection (arrows indicate four unique injection sites within the nTS over time) of SNAP 5114 (1 mM). *Right*: Expanded time traces showing pre-injection (control) and ∼5 min post SNAP injection (denoted by brackets below *left* trace). Note the increase in cardiorespiratory parameters. Injection of vehicle controls had no effect on parameters (not shown). **(B–G)** Group data showing SNAP 5114 significantly increases MAP, HR, SSNA, phrenic amplitude and minute phrenic activity. Phrenic frequency significantly decreased. MAP, HR, SSNA: *N* = 6; PhrNA, *N* = 5, paired *t*-test. Data in **(B–G)** are shown as individual neurons (connected circles), their responses, and means ± SEM in red. *p ≤ 0.05 vs baseline values.

Given the role of GAT-3 on inhibitory and excitatory synaptic signaling we reasoned it would influence cardiorespiratory parameters. To examine this possibility, MAP, HR, SSNA, and PhrNA were monitored prior to and following nanoinjection of SNAP 5114 into the nTS. Representative traces of the hemodynamic, autonomic, and central respiratory responses to unilateral nTS nanoinjection of SNAP 5114 are shown in Figure 6A. As shown, GAT-3 inhibition increased arterial pressure, augmented SSNA, and increased overall PhrNA through an increase in amplitude. Nanoinjection of aCSF had no significant effect on any baseline cardiorespiratory parameters (not shown). Cardiorespiratory parameters prior to and after SNAP 5114 nanoinjection are presented in Figures 6B–G (*N* = 6, *N* = 5 PhrNA). Compared with the preceding baseline, GAT block with SNAP 5114 increased MAP, HR, and SSNA. While PhrNA frequency decreased, the elevation of PhrNA amplitude resulted in an overall increase in minute PhrNA (Figures 6B–G, *p* ≤ 0.05, paired *t*-test vs Bsl).

## Discussion

**FIGURE 7:**
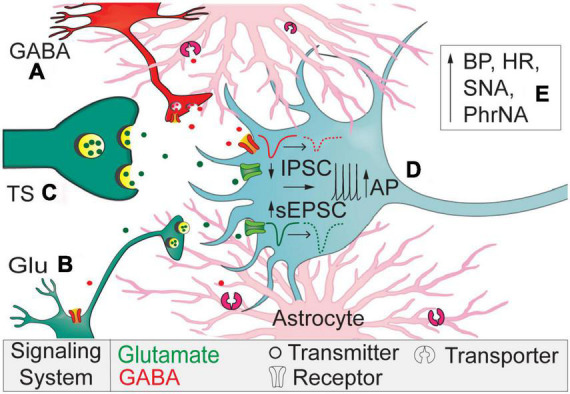
Working model. Astrocytic GAT-3 modulates extracellular GABA and GABA and Glu signaling. **(A)** Block of GAT-3 reduces interneuron somal activity or its release of GABA *via* autoreceptor activation. **(B)** Reduced GABA release disinhibits sEPSCs originating from higher-order neurons to elevate current frequency and amplitude. **(C)** The lack of effect of SNAP 5114 on TS-EPSCs suggest minimal expression of GAT-3 near primary afferents compared to higher-order neurons and synapses. **(D)** The reduced IPSCs and augmented sEPSCs depolarizes nTS second-order somas to increase discharge. **(E)** Overall, GAT-3 inhibition enhances BP, HR, sympathetic, and phrenic nerve activity.

In the present study we demonstrate astrocytic GAT-3 within the nTS tonically affects both inhibitory and excitatory synaptic transmission. Specifically, GAT-3 blockade inhibited GABAergic spontaneous and nTS-evoked IPSCs yet enhanced glutamatergic sEPSCs and discharge. Such divergent responses suggest an interplay in inhibitory and excitatory synaptic signaling that is controlled by astrocytes. These actions ultimately increased MAP, HR, sympathetic, and minute phrenic nerve activity. Figure 7 represents our working model.

The contribution of GABA in synaptic signaling within the nTS is well established (Durgam et al., 1999; Mei et al., 2003; Tolstykh et al., 2003; Jin et al., 2004; Bailey et al., 2008; Fernandes et al., 2011). GABAergic interneurons as well as GABA_*A*_ and GABA_*B*_ receptors are abundant within the nTS (Fong et al., 2005). We expand these studies to demonstrate within the nTS the presence of GAT-1 and GAT-3, the primary GATs within the central nervous system that are encoded by the genes *SLC6A1* and *SLC6A11*, respectively (Zhou et al., 2012). While GAT-1 and -3 are both found within the nTS, GAT-3 expression is greater at the mRNA and protein level. Immunohistochemistry provided the anatomical confirmation that GAT-1 and -3 are expressed in the nTS, and GAT-3 co-labeled with S100B-labeled astrocytes and surrounded GAD67-labeled GABA terminals. Such expression is similar to that found throughout the brain including the cerebral cortex (Minelli et al., 1996), paraventricular nucleus (Park et al., 2009), inferior colliculus (Ghirardini et al., 2018), and basal ganglia (Jin et al., 2011). Based on greater GAT-3 expression, we focused on its neurophysiological and physiological roles within the nTS.

Extracellular GABA is temporally and spatially regulated *via* GATs, including astrocytic GAT-3 that likely contributes to the function of the nTS tripartite synapse. In the present study, the increase in GABA by GAT-3 block attenuated the amplitude of nTS-evoked as well as frequency of spontaneous IPSCs, suggesting GABA-mediated inhibition of GABAergic signaling. Our data are consistent with those illustrating block of GATs depresses evoked and/or spontaneous IPSCs in the neocortex (Keros and Hablitz, 2005), hippocampus (Matos et al., 2018), basal ganglia, and other CNS nuclei (Jin et al., 2011). We and others have shown these IPSCs are eliminated by application of gabazine, the GABA_*A*_ receptor blocker (McDougall and Andresen, 2012; Ostrowski et al., 2014), suggesting GAT-3 limited activation of these ionotropic receptors. The decline in spontaneous current rate suggests the attenuation of GABA signaling is due to a reduction in presynaptic GABA release or attenuation in GABAergic neuron activity. Because GABA receptor blockade eliminates the ability to record IPSCs under these conditions, examination of GABA interneuron discharge in response to GAT manipulation is needed. Additionally, the decrease in stimulation evoked currents suggests attenuated pre-synaptic vesicle release that likely is due to GABA autoreceptor activation (e.g., GABA_*A*_, GABA_*B*_, or GABA_*C*_ receptors), which remains to be determined (Figure 7A). The increase in the IPSC paired pulse ratio with no effect on the IPSC decay with GAT-3 block further suggests presynaptic changes in GABA release.

Gamma-aminobutyric acid transporter 3 inhibition also increased glutamatergic excitation, albeit this occurred primarily *via* changes in spontaneous release. GAT-3 block modestly increased the frequency of spontaneous events within 5 min and progressively increased sEPSC amplitude, counter to our initial expectation that glutamatergic events would decrease with elevated GABA. For instance, activation of GABA_*B*_ receptors on sensory afferents in the nTS decreases EPSCs amplitude (Fawley et al., 2011). One may speculate that the disinhibition due to reducing IPSCs is greater than the inhibition of glutamatergic neurons due to increasing ambient GABA (Figure 7B). The progressive increase in spontaneous EPSC amplitude coincides with the observation that the spontaneous IPSC reduction was not readily removed during our limited 5-min washout period and thus may allow “accumulation” of effects on sEPSCs. Alternatively, in the hippocampus activation of GAT-3 reduces EPSCs *via* heterosynaptic depression. In this process, astrocytic activation of GAT-3 and its coincident elevation of sodium results in an increase in calcium *via* sodium/calcium exchange. Increased astrocytic calcium then induces adenosine release and a reduction of EPSCs (Boddum et al., 2016). While not examined in the present study, if a similar mechanism occurs in the nTS, GAT-3 block may induce disinhibition and thus increase excitation.

The increase in sEPSCs is reminiscent of the similar increase in events following EAAT block, effects that we demonstrated were due to depolarization of nTS neurons and increased tetrodotoxin-sensitive APd (Martinez et al., 2020a). Although not as robust as that which occurs in EAAT block, we observed a similar depolarization of membrane potential and increase in discharge in response to current-evoked depolarization following GAT-3 block (Figure 7D). We also observed a depolarizing shift in holding currents following GAT-3 block, suggesting a basal GABA tone influences excitability perhaps through the above noted mechanisms. In contrast to sEPSCs, GAT-3 inhibition did not alter the amplitude of TS-evoked EPSCs evoked at two frequencies, indicating that at the initial sensory afferent-nTS synapse astrocytic GAT-3 has limited influence (Figure 7C). This is interesting given the fact GABA_*B*_ receptor activation decreases EPSCs (Fawley et al., 2011) and EPSC-IPSC sequences are commonly observed in nTS neurons following TS stimulation (Miles, 1986; Mifflin and Felder, 1988), suggesting GABAergic influence on afferent processing. Such dichotomy of responses in spontaneous and evoked release in the present study may suggest unique localization of astrocyte transporters at the nTS neuropil and afferent synapse. Furthermore, unitary GABAergic inputs, relative to the afferent input, to nTS neurons are sparse and unreliable (McDougall and Andresen, 2012) and thus require convergence of several inputs to influence EPSCs. While we used a minimal stimulation current to evoke nTS-IPSCs, it is possible we generated compound IPSCs based on the size of our electrode. Nevertheless, perhaps more bulk stimulation, or changes in afferent release probability, is required to induce greater GAT influence. In addition, the role of GAT-1 alone or together with GAT-3 at this afferent-nTS synapse remains to be determined, and thus dual blockade may be needed to observe the full influence of astrocyte GATs.

Altogether, our studies support and further extend the notion of glutamate and GABA cross-talk in the nTS. This may occur through activation of ionotropic and metabotropic receptors. For instance, glutamate released from sensory afferents may bind to group II/III metabotropic glutamate receptors (mGluRs) on GABA interneurons to reduce GABA release (Chen and Bonham, 2005) and sIPSC frequency (Jin et al., 2004). Glutamate may also activate GABA neurons *via* Group I mGluRs to enhance GABA release; events that are unique at the initial but not higher order nTS synapse (Fernandes et al., 2011). As we show, such cross-talk also includes GABA modulation of glutamate signaling that is endogenously controlled by astrocytic GAT-3. However, it is also possible that the decrease in sIPSC frequency following GAT-3 block could be modulated through the increase in sEPSC and activation of mGluRs to decrease sIPSC rate, especially within sites adjacent to our recorded neuron. Thus, the ultimate magnitude of GABA contribution to nTS signaling will depend upon its level of release, the location of action, and as we show in the present study, the degree to which GATs are involved and their relative effects on glutamate and GABA signaling.

The nTS is crucial to basal and reflex cardiorespiratory regulation, and GABA plays an important role. Activation of GABA receptors within the nTS increases HR, MAP, and sympathetic activity (Catelli et al., 1987; Mifflin, 2001; Mueller and Hasser, 2006) and reduces respiration (Wasserman et al., 2002) yet these latter responses may be variable and state-dependent (Chung et al., 2006). Conversely, antagonism of GABA receptors lowers blood pressure to indicate that the actions of GABA are a tonic process within the nTS (Catelli et al., 1987; Sved and Sved, 1990). GATs likely contribute to this tonic modulation as their general inhibition with nipecotic acid increased arterial pressure. In the current study, overall nTS astrocytic GAT-3 block with SNAP 5114 elevated pressure, HR, sympathetic nerve activity, and amplitude of phrenic nerve activity while decreasing phrenic rate (Figure 7E), effects likely due to increased endogenous nTS GABA concentrations (Jin et al., 2011). These data indicate that GABA uptake by nTS GAT-3 buffers the inhibition due to nTS GABA and this effect is tonically active in the whole animal. Interestingly, while our results suggest GAT-3 block produces GABA inhibition within the nTS baroreflex circuits, our electrophysiological data indicate effects on both inhibitory and excitatory transmission, ultimately reducing inhibition. nTS function is determined in large part by the balance of excitation and inhibition; GAT modulation of this balance may result in a net buffering of inhibition in the anesthetized animal, which may vary in different states. Alternatively, the variation in autonomic and respiratory effects of GAT blockade suggests distinct effects on different nTS neuronal populations and their projection sites. We bilaterally nanoinjected SNAP 5114 with the goal of achieving global nTS GAT-3 inhibition. The subnuclei affected include neurons that receive baroreceptor and chemoreceptor input as well as neurons that project directly to the pre-sympathetic and respiratory neurons in the ventrolateral medulla (Kline et al., 2010; Alheid et al., 2011). The elevation of sympathetic and phrenic nerve activity following GAT-3 block is consistent with excitation of these non-baroafferent pathways. Further specific examination of individual reflex arcs and nTS neurons with specific projections is needed to resolve this question.

## Conclusion

In summary, we show astrocytic GAT-3 regulates excitation and inhibition within the nTS, with transporter inhibition shifting synaptic signaling toward excitation. Such changes at the circuit level ultimately influence blood pressure, sympathetic, and phrenic nerve activity through one or more autonomic and respiratory pathways that are integrated and relay through the nTS. It is also likely that within the nTS there are additional inputs that originate upstream that are influenced by GATs, for instance those GABAergic circuits from the amygdala or periaqueductal gray that influence autonomic tone (Saha, 2005; Netzer et al., 2009). As our previous studies demonstrating changes in glutamate transporter expression and/or function contribute to nTS adjustments in hypoxia (Martinez et al., 2020a; Matott et al., 2020), one may speculate alterations in GAT-3 will have an influence on nTS circuits and the associated cardiorespiratory parameters in physiological stressors or disease. Nevertheless, these studies confirm the importance of astrocytes to neuronal and synaptic function.

## Data Availability Statement

The raw data supporting the conclusions of this article will be made available by the authors, without undue reservation.

## Ethics Statement

The animal study was reviewed and approved by the University of Missouri Animal Care and Use Committee.

## Author Contributions

EH and DK conceived and designed the experiments. MM and DM performed the IPSC recordings. LL-S performed the EPSC and discharge recordings. EH performed the *in vivo* experiments. All authors analyzed and interpreted the data. DK wrote the manuscript.

## Conflict of Interest

The authors declare that the research was conducted in the absence of any commercial or financial relationships that could be construed as a potential conflict of interest.

## Publisher’s Note

All claims expressed in this article are solely those of the authors and do not necessarily represent those of their affiliated organizations, or those of the publisher, the editors and the reviewers. Any product that may be evaluated in this article, or claim that may be made by its manufacturer, is not guaranteed or endorsed by the publisher.
